# Development and evaluation of large-language models (LLMs) for oncology: A scoping review

**DOI:** 10.1371/journal.pdig.0000980

**Published:** 2025-08-07

**Authors:** Namya Mehan, Teshan Dias Desinghe, Ashirbani Saha

**Affiliations:** 1 Integrated Biomedical Engineering and Health Sciences, McMaster University, Hamilton, Ontario, Canada; 2 Global Health Program, Faculty of Health Sciences, McMaster University, Hamilton, Ontario, Canada; 3 Department of Oncology, McMaster University, Hamilton, Ontario, Canada; 4 Escarpment Cancer Research Institute, McMaster University and Hamilton Health Sciences, Hamilton, Ontario, Canada; 5 Centre for Data Science and Digital Health, Hamilton Health Sciences, Hamilton, Ontario, Canada; Massachusetts Institute of Technology, UNITED STATES OF AMERICA

## Abstract

Large language models (LLMs), a significant development in artificial intelligence (AI), are continuing to demonstrate seminal improvement in performance for various text analysis and generation tasks. There are limited systematic studies on LLM applications that were developed/evaluated in relevance to oncology. Our scoping review explores applications of LLMs in oncology to determine (1) the nature of LLM applications relevant to a cancer/tumor type, (2) the phases of cancer care addressed by the LLMs, (3) which LLMs were used in these applications, (4) the sources and pre-processing of datasets used, (5) the techniques used to optimize the performance of LLMs, (6) the methods of evaluation, and (7) the common limitations noted by the authors of these LLM applications and to study their implications in research and practice. A librarian-assisted search was performed across the following databases: Association for Computing Machinery (ACM), Embase, Engineering Village, IEEE Xplore, Medline, Scopus, SPIE and Web of Science till Jan 12, 2024. Pre-prints from this search were considered if they were published/accepted by Feb 29, 2024. From the initial search of 14863 articles, 60 were finally included. Our results demonstrated that LLMs were mostly evaluated across a diverse set of oncology-related applications. Generative pre-trained transformer (GPT)-based LLMs were mostly used. In the subset of studies where the phase(s) of cancer care was/were provided or implied, treatment and diagnosis were the most included phases. Data for development and evaluation extended from patient health records, synthetic patient records, research and professional society publications to social media. Prompt-designing and engineering were performed as data pre-processing steps in several studies. Clinicians, trainees, researchers, and patients were among the variety of users targeted by the applications. In the17% studies that developed LLMs for oncological aspects, domain adaptation through pre-training and fine-tuning were often performed and resulted in performance improvement. The evaluation of an LLM’s performance involved usage of both standard, validated, non-standardized, and/or customized performance measures considering a variety of constructs, other than accuracy. Six primary themes emerged as limitations including limitation of generalizability/applicability, sample size, bias and subjectivity, and evaluation metrics. This review highlights that LLMs, specific to oncological aspects, are less common than general-purpose LLMs. The application areas were heterogeneous, used diverse data sources, were directed towards a variety of users, and resulted in variety of evaluation methods. Despite the diversity of LLM applications in oncology, future research needs to address the limited generalizability of these applications, mitigation of bias and subjectivity, and standardization of evaluation methodologies. Future applications of LLMs in oncology should include developing oncology-specific LLMs that can mitigate knowledge gaps and extend to diverse areas of oncology training and practice not considered so far.

## Author summary

Large Language Models (LLMs) have emerged in the past few years as a groundbreaking technology in natural language processing and artificial intelligence. Given the rapid proliferation of research studies using these models, our team wanted to broadly scope out the different applications related to these models in oncology. We found that LLMs, especially generative pre-trained transformer models, are the most used ones. Most LLMs were used off-the-shelf with the help of prompting. Few LLMs were developed or adapted for the application. LLMs were used across a variety of areas in the cancer care continuum including prevention, screening, diagnosis, and treatment as well as in training and education on cancer. These models worked on various data sources, from patient records to social media, aiming to help different stakeholders such as doctors, researchers, and patients. However, limitations of these applications exist including restricted applicability in small datasets, bias, and lack of standard evaluation methods. Future efforts should focus on creating cancer-specific LLMs and addressing these limitations.

## Introduction

### Background and rationale

Language models (LMs) are computational systems designed to understand and produce human language. Their development has progressed through several stages, including statistical language models, neural language models, and pre-trained language models, ultimately leading to the creation of large language models (LLMs) [1]. LLMs are built using artificial neural networks with a massive number of parameters to the order of several millions, billions, and even trillions. They are noted for their remarkable abilities to apparently understand, analyze, and/or generate general-purpose human text [2,3]. LLMs that empower technology for text generation are one of the major accomplishments in the ongoing generative artificial intelligence (AI)-related work [4,5]. LLMs are significantly impacting diverse disciplines and areas, including medicine in general [6], radiology [7], and oncology [8]. This has resulted in several recent studies (published or archived) that reviewed or surveyed LLMs in the field of medicine, as listed in the study by Zheng et al. [9].

In relation to cancer or oncology, numerous studies investigated the use of LLMs and LLM-based AI tools for further development and evaluation. This includes using the LLMs as-is (also called off-the-shelf/out-of-the-box) for evaluation, or developing or adapting general-purpose LLMs by exposing those to oncology-relevant data (domain-adapted) and making those more cancer-specific. As of now, there are limited systematic studies [8,10] on LLMs and oncology. Iannantuono et al. [8] covers a limited number of published works (PubMed articles till July 12, 2023) for oncology. Sorin et al.’s study [10] is limited to only breast cancer-related studies using LLMs till October 22, 2023. A more recent systematic review and meta-analysis study by Carl et al. [11] exclusively used PubMed as a database source, limiting the number of studies that could be considered. Therefore, the need for a comprehensive analysis resulting in an overview of the usage and necessity of different types of LLMs (both general purpose and oncology-specific) in oncology exists. A scoping review, as per the recommendations of Arksey and O’Malley [12], can address this gap by summarizing a large body of literature generated within a short span of time to derive an overview of the evidence.

Therefore, we performed a scoping review on studies developing/evaluating applications relevant to oncology. The specific goal of this review is to answer questions related to – the type of applications powered by LLMs, cancer types involved, users of LLM-based applications, phases of cancer care, data source and preparation methodologies, sample sizes and demographics, types of LLMs used, optimization of the used LLMs and its effects on the performance, evaluation methodologies, and limitations noted by authors. We concluded finally by reflecting on the implications of using LLMs in research and practice for oncology.

## Results

### Selection of the sources of evidence

**Fig 1:**
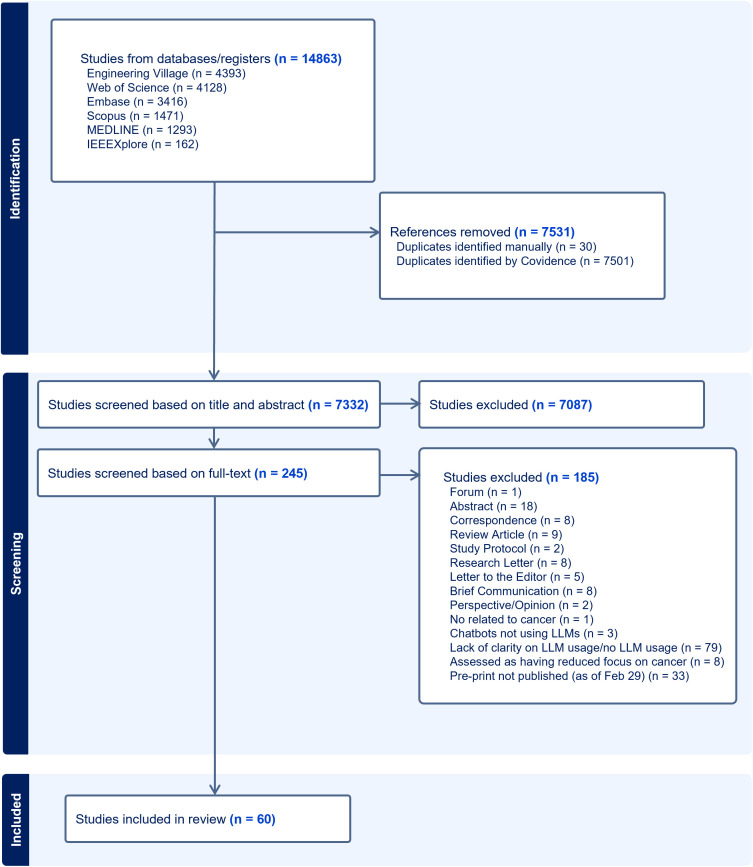
The PRISMA diagram relevant to our study.

Our initial search resulted in 14863 articles. Our search strategy included prompts and general terms for LLMs and oncology such as “chatbots”, “LLM”, “generative AI”, “cancer”, “tumours”, “oncology” and “medical oncology” (Full search strategy located in S1 Data). Post-screening (based on our inclusion and exclusion criteria), our study included 60 articles as the source of evidence (PRISMA diagram in Fig 1). The data charted is provided in S1 Data (tab: ‘Data Charted’).

### What types of applications are powered by LLMs?

Eighty-three percent (50/60) of the studies performed evaluation of the off-the-shelf LLMs on a variety of applications or used a pre-trained LLM-based feature representation in their work. Development and/or adaptation of LLMs were performed in 10 studies (S1 Data spreadsheet, tab: ‘Data Charted’).

Evaluating the ability of LLMs to generate responses to specific inputs or queries [13–37] was performed in 42% (25/60) of the studies. These specific queries included questions related to information for patients and/or caregivers (such as frequently or commonly asked questions by common people), simplification of content for common people, and questions related to trainees/experts-in training (examination questions).

In 30% (18/60) of the studies [21,23,26,30,32,35,38–49], LLMs were used for generating recommendations for possible imaging procedures, treatment and follow-up plan, medical tests, CPS genes, and decision-making based on patient-specific clinical scenarios, patient profiles, case vignettes, or case reports presented.

Information mining methodologies were developed/evaluated in 18% (11/60) of the studies [50–60] using clinical or test reports, medical documents, and social media. Summarization of text in reports was conducted by 8% (5/60) of the studies [16,61–64].

In two studies, the role of LLM-based chatbots to elicit responses from patients on their condition/history [64,65] was evaluated. Other applications included natural language inference in clinical trial reports [66], supporting clinical scientists (in the context of one the studies, all were trainee or consulting physicians performing scientific research) with scientific tasks, such as academic/scientific research or writing [67,68], classification of reports into pre-defined categories [57,64,69,70], classification of drug-pair synergy [71], and database construction [72].

**Fig 2:**
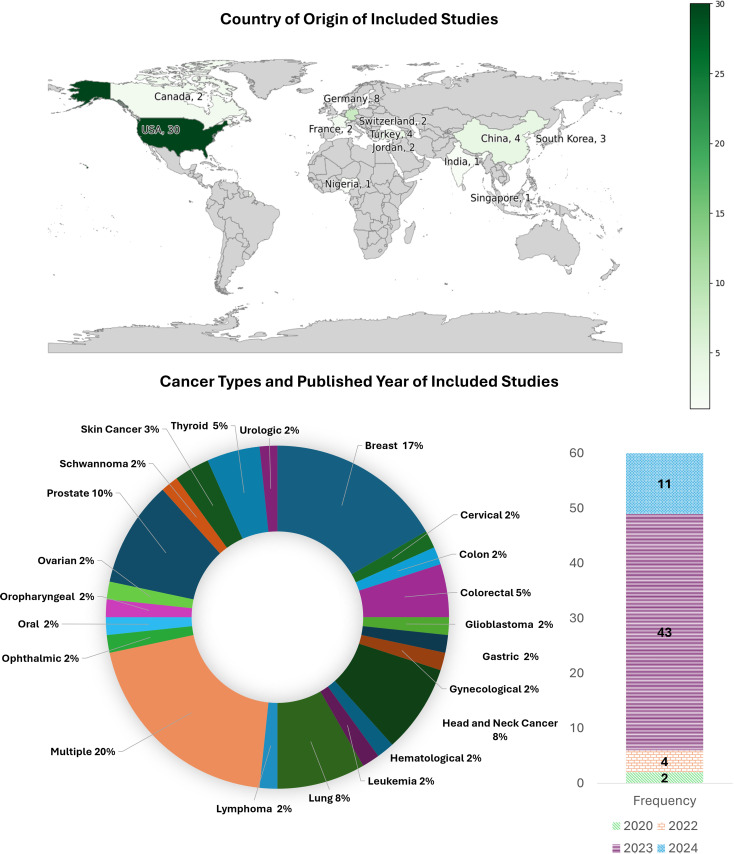
Cancer types, year-wise frequency, and countries of the included studies. Basemap obtained from https://www.naturalearthdata.com/downloads/110m-cultural-vectors/110m-admin-0-countries/.

The distributions of the studies across the cancer types, publication year, and country of publication are shown in Fig 2. Users of the applications were identified as: patients or lay people (14/60), healthcare providers (13/60), researchers (3/60), both healthcare providers and patients (14/60), healthcare providers and other users (researchers and/or medical educators and medical trainees, 16/60).

To summarize the findings for this question, the LLMs have powered a variety of applications relevant to oncology. In most of these applications, LLMs were used off-the-shelf and not adapted. Potential users of these applications were identified as healthcare providers and trainees, educators, researchers, patients, and common people.

### Which phases of cancer care are mostly addressed by LLMs?

For 40% of the studies, the targeted phases of cancer care were not apparent or likely to include many phases of the cancer care continuum. As shown in Table 1, the studies could relate to more than one phase of the continuum, or be non-specific about the phase, or be specific to one phase.

**Table 1:** Different phases of cancer care supported by the studies.

Phase of cancer care*	Number of studies	References
Prevention	6	[18,29,33,45,47,48]
Screening	8	[17,18,26,29,30,45,49,53]
Diagnosis	12	[24,30,32,33,48,51,53]
Treatment	17	[24,25,32,33,38,42–44,46–48,51,64,71]
Survivorship	9	[39–41,52,64,72]
End-of-life	2	[39,40]
Non-Specified/Several (more than 3)	24	[13–16,19–23,27,28,31,34–37,55,56,59,61–63,67,68]

*Studies supporting up-to three phases are shown in individual phases, otherwise, they are mentioned in the Non-Specified/Several (more than 3) category.

To summarize the answer to this question, treatment-related studies (17) were more common than other phases, followed by diagnosis (12).

### Why and which LLMs (their characteristics) are being employed for development and evaluation tasks in oncology?

Majority (40/60) of the studies reported using a single LLM or a single version of an LLM. Twenty (20) studies used more than one LLMs in their application(s). Generative pre-trained transformer (GPT)-based models such as GPT2 [73], GPT3.5, GPT4 and the versions used in ChatGPT (decoder only, ranging from a several millions to several billions of parameters) were applied in the 82% (49/60) of the studies. Among these, 37 studies used GPT-based models only whereas the other 12 used several other LLMs in conjunction with or for comparison. The studies that did not use GPT-based models, used at least one bidirectional encoder representations from transformers (BERT)-based model (encoder-only) apart from one study that used blenderbot-400M-distill (3 billion encoder-decoder, 30 billion, or 175 billion decoder-only), which is based on R2C2 or open-pretrained transformer (OPT) [74] depending on the version selected. A summary indicating the names of LLMs noted in our review and their primary applications are shown in Table 2.

**Table 2:** LLMs and their main uses in the included studies.

LLM Groups or LLMs	Uses noted in our study	Number of studies
BERT & Variants (BERT, RoBERTa-base, RoBERTa-large, BioClinicalBERT, CancerBERT, PathologyBERT, BETO, caBERT, BioBERT, RadBERT, BERT_MIMIC, DeBERTa_MIMIC RoBERTa_MIMIC, KcBERT, KlueBERT, KoBERT)	Text/information mining, Natural Language Inference, Classification, Text Summarization, Providing emotional support as chatbot	12
ChatGPT Variants (ChatGPT 3.5, ChatGPT 4, ChatGPT Plus, ChatGPT Free, ChatGPT June 17, 2023, ChatGPT Jan 9, ChatGPT March 23, 2023) and other GPT-based/style (GPT-2, GPT-3, GPT-3.5, GPT-4, BioMed LM, CancerGPT)	Generating recommendations, Answering queries, Text summarization, Text/information mining, Literature review, Scientific/academic research, Drug pair synergy prediction, eliciting responses, Database construction	49
Google Bard (Bard, LaMDA)	Answering queries, Text simplification, Generating recommendations	3
Other LLMs/LLM-based Chatbots (Galactica, Perplexity.ai, Perplexity {concise and detailed model}, Bing AI, NeevaAI, Chat Sonic, YouChat, BLOOMZ)	Generating recommendations, Answering queries	2
Other LLMs (GatorTron, LongFormer_MIMIC)	Text/information mining	1
Other LLMs/Chatbots (BART, PEGASUS, T5, Clinical-T5, FLAN-T5, BERT2BERT, PGN, OPT, LLaMA-LoRA, Alpaca-LoRA, BioBART)	Text summarization	1
Other LLMs (SciFive)	Drug pair synergy prediction	1
Other LLMs/Chatbots (Blenderbot-400M-distill)	Eliciting responses	1

For selecting ChatGPT, the authors highlighted reasons such as, human-like text generation, providing appropriate and/or quick responses to diverse (generalized and specialized) scenarios, having clinical insights, usage by experts and patients, and the ability to address unseen scenarios. The studies using non-GPT based LLMs mostly mentioned the improved performance of the models in NLP tasks over traditional machine learning or previous versions of LLMs.

To summarize, GPT-based models (including ChatGPT) have been a major driver of the studies that we already know from the answer to the first question were mostly off-the-shelf studies. Together these two answers indicate the technical innovation in model development and benchmarking were less common than LLMs’ off-the-shelf usage.

### How are datasets being acquired and pre-processed for these tasks and what are the demographic characteristics?

Various data sources have been used in the studies for training and/or evaluation. These include the usage of patient health records in specific/across institutions (23/60), including clinical cases from Red journal gray zone, public resources of medical data/research/information prepared by experts/professionals including professional society websites (14/60), guidelines/consensus documents/expert recommendations (11/60), synthetic patient profiles/records (7/60), social media/social pattern/patient support groups/web scraping (6/60), examination question sources (3/60), common/relevant expert knowledge/expertise (2/60), clinical scientists as human subjects (1/60). Three studies were not specific about the source of their data.

The data pre-processing steps included prompt design and engineering [75] as several studies performed evaluation using off-the-shelf LLMs. Some studies reserved a part of their datasets for designing optimal prompts, i.e., prompt design and engineering before evaluating the remaining parts. Some studies also provided a knowledge base with prompts to improve performance [14,45]. A combination of text cleaning, stemming, filtering, splitting, annotation, formatting, text representation was used in studies that developed LLMs. The data input to LLMs could be either raw and cleaned and/or structured.

Of the studies that reported demographic characteristics of the data, the overall age reported included an extensive range of ages for (1.5 years to an upper limit of 95 years) human subjects (real/synthetic, patients/scientists), of both sexes altogether. The users/evaluators/reference-standard generators were mostly doctors belonging to various specialties (e.g., radiation oncologists, dermatologists, radiologists, pathologists, surgeons of different specialties, gynecologists, thyroid specialists), members of tumor board, medical trainees (student/resident/fellows), medical examiners, certified tumor registrars, and of undisclosed expertise in some cases. The expertise or nature of the users/evaluators/reference-standard generators was mentioned in 68% of the studies and 35% provided patient demographics. Studies that developed LLMs needed bigger data samples of various units (e.g., patients, reports, clinical notes, questions, patient profiles) for training or pre-training (if involved) resulting in the usage of larger datasets overall compared to those who evaluated LLMs.

To summarize, data sources were diverse in nature in terms of content and demographics. The pre-processing depended on the type of the application. Evaluators/users or reference standard providers, if disclosed, were most likely have professional or in-training expertise in the content/application.

### What techniques are used to optimize LLMs?

The details of the LLM optimization for the studies (N = 10) that trained (developed) LLMs are presented in Table 3 in relation to relevant tasks for oncology. Altogether, these studies have used both unlabeled data and labeled data with respect to their tasks. These oncology task-specific LLMs were found to be often better than other LLMs or machine learning models in the task of interest. Single cancer type-specific datasets were used by 50% of these studies (breast cancer: 3, thyroid cancer: 1, lymphoma: 1) and the remaining used datasets from a variety of cancers.

**Table 3:** Details of optimizing LLMs by the studies that developed/adapted LLMs and their performance.

Reference	Training/Adaptation/Optimization used for LLM	Database used	Relative performance of the developed LLM(s)
Alissa et al. [66]	New LLM that consists of two RoBERTa-Large models was trained; hyperparameter tuning was performed	Annotated (i.e., labeled) *breast cancer* clinical trial reports from clinicaltrials.gov (training)	New LLM outperformed the BERT and RoBERTa models
Huemann et al. [69]	Domain-adaptation and fine-tuning of pre-trained BERT, RadBERT, RoBERTa, and bioClinicalBERT	A PET/CT dataset containing the word *lymphoma* (adaptation), PET/CT reports having the word Deauville score (fine-tuning)	Domain-adapted BERT performed the best
Kim et. al [72]	Domain adaptive pre-training (knowledge and vocabulary) to KcBERT (a BERT model capable of processing Korean)	A dataset was generated specifically for domain-specific terminologies in Korean: Korean Semantic Textural Similarity (KorSTS) [76] dataset and Korean Emotional Conversation Corpus [77] (with utterances related to *cancer*)	Performance improved with domain-adaptive training
Li et al. [71]	Developed CancerGPT, full-finetuning GPT-2 and subsequent selective (last layer) fine-tuning	Labelled data from DrugComb portal [78] on a *variety of cancer cell lines* for drug combination screening studies using common cancer data first, followed by rare cancer data	CancerGPT performed better than other LLMs and general prediction models
Mitchell et al. [54]	CancerBERT Network: pre-trained ClinicalBERT was re-trained, a question-answer model was developed based on multi-stage, task-based training and fine-tuning	A large corpus of institutional pathology reports (re-training), SQuAD [79], BioASQ [80], dataset from local institutional data labeled for *a variety of tumor sites and histology* (question-answer model)	Models from this network outperformed ClinicalBERT
Nunez et al. [70]	Fine-tuned BERT (BERT-base-uncased)	Labeled survival data on *a variety of cancers*	Traditional models performed better than LLM
Pathak et al. [56]	Four general purpose LLMs were re-trained on clinical notes, GatorTron (BERT-style) trained from the scratch	Clinical notes from MIMIC dataset [81] (re-training general purpose LLMs), local institutional clinical notes (pre-training GatorTron), labeled *thyroid ultrasound report* dataset (fine-tuning)	GatorTron performed better than the other LLMs that were not pre-trained on local data
Santos et al. [57]	PathologyBERT: Pre-training of BERT followed by fine-tuning	Local institutional unlabeled histopathology reports (pre-training), labeled histopathology reports with respect to *breast cancer* (fine-tuning)	PathologyBERT which outperformed ClinicalBERT (pre-trained on notes of diverse diseases) and BlueBERT (pre-trained on Pubmed abstracts and clinical notes from MIMIC)
Tie et al. [63]	Twelve open source LLMs were training and hyper-parameter tuning	PET reports from the local institution	Top-performing LLM (PEGASUS) was found useful by clinicians
Zhou et al. [60]	CancerBERT and its variants: pre-training of BlueBERT to form CancerBERT and fine-tuning	Unlabeled *breast cancer*-related clinical notes and pathology reports (pre-training), labeled *breast cancer* reports (fine-tuning)	CancerBert outperformed domain-specific BERT models

To summarize, the adaptation of pre-trained LLMs is evident in most of the studies that performed development, including already pre-trained LLMs accessed by the author or by pre-training/retraining models of relevant corpus of data. Domain-specific training and adaptation improved the performance of the LLMs in specific tasks.

### What are the methods of performance evaluation?

**Fig 3:**
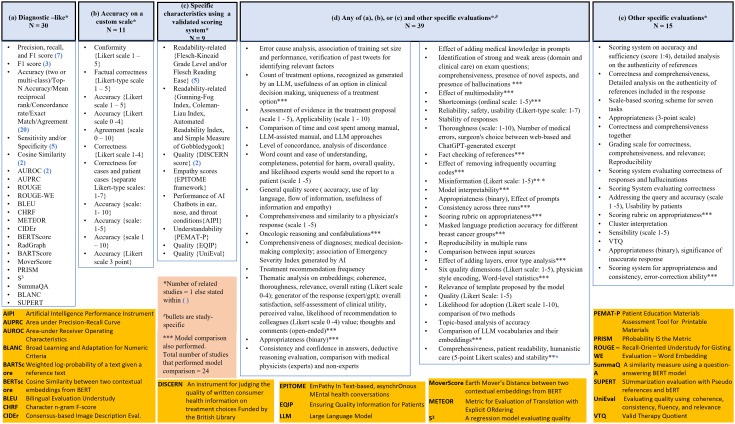
Categorization of different methods of evaluation.

We identified several types of evaluation strategies consisting of quantitative and qualitative approaches. The aggregated information of the evaluation metrics is shown in Fig 3 (further paper-level details are provided in S1 Data spreadsheet, tab: ‘Evaluation-Detailed Analysis’). To present this information, we grouped the evaluation into five categories, centered around the construct of accuracy (refers to the degree to which an output or response matches with reality or a true value), as follows:

(a) Diagnostic-like: In this category, evaluation of accuracy of the outputs was carried out against a pre-set comparator/ground truth/reference standard using different standard performance measures. Examples include studies considering quantitative measures such precision, recall, F1, accuracy [50], sensitivity, specificity [58], AUROC, AUPRC [71].
(b) Accuracy on a custom scale: In this category, evaluation of accuracy was carried out using a custom-scale. Examples include usage of Likert-type scales by Braun et al. [39] for evaluating the conformity of ChatGPT’s response with the guideline and by Koroglu et al. [23] for measuring correctness using human observers/raters.
(c) Specific characteristics based on a validated scoring system: In this category, evaluation of specific characteristics (may or may not involve accuracy) of the output was carried out using existing scoring systems. Examples include evaluation of readability by Chung et al. [61] using Flesh-Kincaid Grade Level scores and evaluation of performance of AI chatbot on a variety of constructs by Lechien et al. [44] using Artificial Intelligence Performance Instrument (AIPI).
(d) Any of the (a), (b), or (c) and other specific evaluations: In this category, additional evaluations were performed on top of performing evaluations related to any of (a), (b), (c). Examples include: the study by Davis et al. [14] where comprehensiveness and similarity to a physician’s response were evaluated using a scale, in addition to measuring accuracy with a Likert scale and the study by Huemann et al. [69] where a comparison among models and an assessment of the effect of multimodality were performed in addition to measuring five-class accuracy.
(e) Other specific evaluations: In this category, other specific evaluations were carried out only. Examples include Rahsepar et al.’s study [29] that developed a scoring system on accuracy and sufficiency together and Schulte et al.’s [47] evaluation of the output using valid therapy quotient (VTQ) measuring the proportion of acceptable recommendations.

Some evaluation considered resource utilization such as latency [65], time and cost-effectiveness [51] and response speed of the Chatbot [37]. All studies (N = 10) that developed LLMs performed model comparison (i.e., tested the developed LLM(s) against other LLMs or machine learning models). Only 13 studies that performed evaluation did a model comparison. Several studies also performed assessment of inter-rater agreement when human reviewers were needed in the scoring process [13,19,23–27,32,34,37,43,50,52,63,68].

To summarize, the studies demonstrated heterogeneity in their evaluation. This is expected and understandable as LLMs were used in a variety of new applications and that required ingenuity to evaluate the performance. The evaluation measures are also informed by common errors exhibited by the LLMs such as hallucination, bias, and lack of stability of responses.

### What are the limitations noted by the authors and are there commonalities in the limitations?

**Fig 4:**
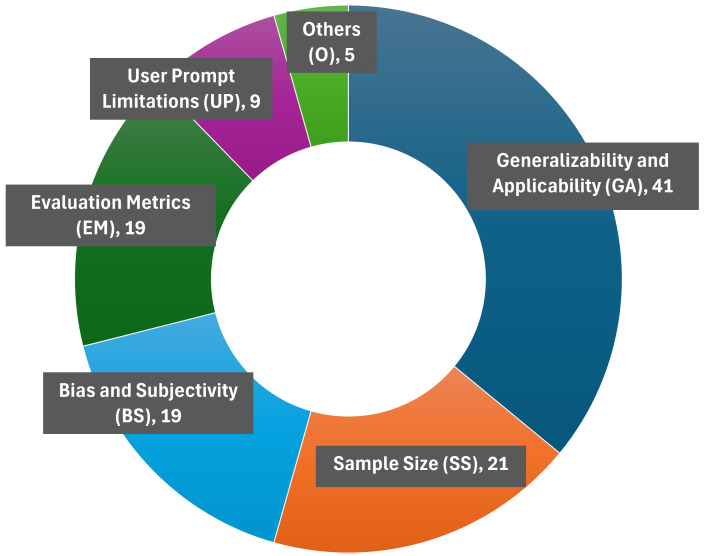
Six themes identified from the author-stated limitations.

A thematic analysis reveals the presence of six key limitation themes including *generalizability and applicability, sample size, limitations concerning evaluation metrics, bias and subjectivity, user prompt limitations and other concerns.* The key themes and corresponding sub-themes are noted in Fig 4 (comprehensive list in S1 Data spreadsheet, tab: ‘Thematic Analysis’). Out of the six themes, the most common limitation discussed across papers was the *generalizability and applicability* (68.5%) of study findings. This theme included issues stemming from the inclusion of synthetic test data [38] and fictional cases [39], incorporating a homogenous group of patients [52] or single institutional studies [25,42,56,60,61,63,69]. Study design limitations such as the absence of control groups and accuracy issues caused by data extrapolation [51] and overfitting [62] were also considered in this theme.

The theme of *sample size* (35%) was the next most common theme including limited training (includes contextual information in prompt design as well as authors often consider that training) data [33,37] and small sample size (low number of clinical cases [44] and small set of questions [29]) as sub-themes. Small sample size (26.7%) is the most identified sub-theme in the study.

Limitations resulting from *evaluation metrics* (31.8%) including inaccurate metrics [17], limited validation procedures [52,56,70], rating discrepancies [19,21], and [17], unverified and incomplete metrics [13] was the next frequent theme. Additionally, limitations stemming from *bias and subjectivity* (31.6%) from implicit knowledge [17,62], subjective questionnaires [34], bias in evaluation process and scoring systems [19] were also discussed.

*User Prompt Limitations* (15%) including formulation of queries [13], lack of question comprehensiveness [17], and lack of standardization method to optimize prompts [45], and *other* (8.3%) concerns such as human error [26,51] in conducting the studies and resource constraints [21,62,63] were also outlined.

Given that most included papers outlined inherent limitations of LLMs, author-stated LLM limitations were excluded from analysis, e.g., lack of updated knowledge, variation resulting from multiple inputs and in response latency, rapid LLM evolution, and lack of updated model parameters.

To summarize, a thematic analysis was conducted on the author-stated limitations of the studies. Generalizability and applicability were the main limitations across the studies. This was expected due to the limited scope of individual studies and lack of available generalized oncology datasets for testing LLMs. In addition, sample size, bias and subjectivity, and evaluation metrics were also common limitations.

### Secondary research questions

Our study did not find genetic counseling to be a dominant application of LLMs in oncology. Most applications were developed or evaluated for text processing in the English language. Other languages include Chinese [53], Korean [72], Spanish [58], and German [42,46]. Equity and socioeconomic differences were not evaluated. One study [14] mentioned this in the context of human papilloma virus-positive and negative oropharyngeal cancer patients and another study [65] mentioned this as a limitation considering racial, cultural, gender, and ethnic biases.

## Discussion

### Main outcomes

This is a timely scoping review of the dynamic field of AI and oncology with a focus on large language models (LLMs). The concept of LLMs is relatively new (2018) but even newer is ChatGPT (2022) which is the fastest adopted application in human history [82,83]. It generated significant interest in a variety of fields including medicine [6] and its sub-field, oncology. ChatGPT is powered by an LLM, so it is important to scope out the impact of LLMs on oncology.

We found that most LLM applications were related to English-language texts, based on off-the-shelf-usage of general-domain-based (as opposed to becoming more domain-specific through CancerBERT, CancerGPT, PathologyBERT) LLMs or chatbots, and were related to eliciting responses to specific queries or generating recommendation. The seminal usage of general purpose LLMs implies that the development of oncology-specific LLMs is relatively low (in 17% of studies only and an exception is CancerLLM [84] which is an archived study considering our cutoff date and therefore, not included) and highlights an important research gap. Despite the LLMs’ ability to generalize to a variety of domains, non-medical LLMs can exhibit significant knowledge gaps [85]. This is noted in our analysis as most of the studies found LLMs trained/adapted for specific tasks to improve performance. Furthermore, studies engaging in LLM development examined a limited number of oncological applications. As of now, generalized usage of a single oncology-specific LLM throughout the various aspects (e.g., clinical diagnosis and care planning, assessments and documentation, communication with patients, evidence summarization, education, research and collaboration) [86–89] of oncology practice and training, even for a single type of cancer, is limited.

Our study revealed that there has been greater research interest in applying LLMs for diagnosis and treatment phases of the cancer care continuum than the other phases. For diagnosis and treatment, studies on single cancer type were more prominent. However, there were studies involving multiple tumors based on the type of application. Most phases of breast cancer and lung cancer were addressed by considering different studies together. Several phases of cancer care for prostate, head and neck, skin, and thyroid cancers were addressed. In studies that revealed type(s) of cancer and phase of cancer care, breast cancer screening, diagnosis, and treatment were found to be of significant interest. This highlights that there remain many cancer types (including rare ones) to be addressed by LLM-based applications across the cancer care continuum. This also highlights that in terms of reporting, studies could be more transparent on the type(s) of cancer being studied.

The most prominent family (the family refers to the base architecture of the model) of LLMs [90] used was GPT (a decoder only model architecture developed by OpenAI), followed by BERT (an encoder only model architecture developed by Google). While earlier GPT-based models (GPT-1 and 2) are open source, recent GPT models (including the models that empower ChatGPT) are closed source at this point. In comparison, BERT is an open-source (both source code and pre-trained model are available). The benefit of having an open-source model is that it can be re-trained or fine-tuned or even trained from scratch in various tasks, including different data sources, and potentially improved (e.g., reduced bias). While the papers using BERT-based models mentioned the superior performance of BERT in NLP tasks or pointed out improved BERT-based versions, studies using GPT-based models stated various specific abilities of GPT-based models in a more diverse set of tasks. As determined by results, GPT-based studies were more focused on evaluation, whereas BERT-dependent models often involved development and/or adaptation. The other families of LLMs, such as LLaMA, T5, were used in some studies. It is likely that more models (including, multimodal) will be developed and evaluated based on new and emerging LLMs in the future.

The pre-processing of data predominantly involved designing prompts. Studies that performed development or used pre-trained LLMs in their applications used other relevant pre-processing. Depending on the application, the type of data sample (i.e., model input) changed as they emerged from a variety of sources (e.g., guidelines, examination questions, public sources of medical data) other than patient data (i.e., from patient health records or synthetic patient data). Therefore, demographic details did not apply to all datasets used in development/evaluation. Rather, demographic details were more relevant to prompt developers, ground truth developers, users, and evaluators of the application as these involved humans. Several studies reported the qualifications of ground truth developers. It is important to understand the demographic characteristics of human evaluators or users to improve the reliability of a study unless the ground truth is already derived from the documents of trusted professional societies or qualified working groups.

Optimization of LLMs involved re-training pre-trained LLMs on relevant medical corpus, fine-tuning, and hyperparameter tuning. As our review found a smaller number of studies that have developed LLMs, a limited variety of optimization techniques is understandable, but optimization led to improvement in performance in specific-tasks mostly in terms of accuracy-related performance measures. The studies reported diverse methods for evaluating LLMs’ performance. Given the diverse nature of applications and lack of standard evaluation protocols for evaluating the performance of the LLMs, this is understandable. Typically, the task of LLM evaluation is established as a challenging one. It includes the practice of evaluating LLMs across a variety of performance benchmarks for different characteristics [91]. However, similar established benchmarks are rare in oncology. Considering the environment in which the LLMs are applied, there is a need for developing strong benchmarks in oncology where emerging LLMs or applications can be tested for a variety of essential characteristics, including specific measures of clinical accuracy, safety, and outcome impact.

Our study found commonalities in the limitations discussed by the authors in addition to the existing limitations of the LLMs. The prominent ones include generalizability and applicability, limitations concerning evaluation metrics, sample size, bias and subjectivity, and user prompt limitations. Given the early stage of research involving LLMs in oncology, such limitations are likely but expected to be mitigated in the future. Furthermore, through these limitations, a seminal responsibility of using LLMs for oncology is highlighted, particularly when applying those on human subject data in the context of automatic decision-making. Overall, our study determines heterogeneous applications of oncology and the promise of those align with the literature that discusses incorporation of LLMs in healthcare, under mitigated risk, as an ethical necessity in accordance with principlism [92]. At the same time, the needs of human oversight [93] for their application and tailored governance [94] are also supported by the author-stated limitations of the studies we analyzed. Our findings also highlight the lack of ethnic, linguistic, and geographical diversity in the data (could be caused due to reporting) and applications. This further illustrates the need for systematic studies to evaluate the applicability of LLMs in oncology by also considering equity (e.g., low health literacy, scarcity of digital resources, low and middle-income countries) and diversity (e.g., various cultures, subjective biases).

### Limitations

Our scoping review has some limitations. We are restricted by the search end dates and keywords related to this dynamically evolving field. There remains a possibility of missing publications (such as [95]) as we report no articles published in 2021 or after the cut-off date related to LLMs. Our choice of keywords may have resulted in the retrieval of more articles related to ChatGPT/GPT and less articles in the favor of other LLMs, particularly if the model(s) used are not identified in the respective publications as LLM(s) but as an advanced natural language processing model. Additionally, our study is restricted to publications written in English and therefore, may cause bias to the language related findings we noted. Further, a critical appraisal of the sources of evidence was not performed, which may have had an impact on our findings.

### Conclusions

This review highlights that LLMs, specific to oncological aspects, are less common than general-purpose LLMs. The application areas were heterogeneous, used diverse data sources, and supported a variety of users. Despite the diversity of LLM applications in oncology, future research needs to address the limited generalizability of the findings of these applications, presence of bias and subjectivity, lack of standardization of evaluation methodologies, and lack of equity and diversity related research design and reporting.

This study raises several key implications for research and practice. This includes the development and evaluation of oncology-specific LLMs that could apply to a variety of cancers and across a variety of tasks, and therefore, would be more generalizable within the field of oncology. It also underscores the need to develop appropriate evaluation metrics for different sets of tasks. There exists a gap in applying LLMs to an extensive set of tasks relevant in daily or usual oncological practice or in practices (e.g., radiology, pathology) that partially overlap with it. However, there could be limited scenarios in oncology where use of LLMs could be conducted more rigorously to accomplish their utility and potentially pave a way of improving efficiency. Since most studies included in this review focus on the evaluation of LLMs for use in cancer, the need for standardized measures to evaluate the LLMs in different clinical scenarios is highlighted. This scoping review also underlines the lack of discussion on the LLMs’ consideration of patient socioeconomic differences, inequities and bias resulting from training data which would prove to be relevant to their use in clinical practice.

## Materials and methods

### Study framework, protocol, and reporting

The review followed the framework proposed by Arksey and O’Malley [12]. The review protocol was registered at https://osf.io/kmvrf/?view_only=d3ca5c704843498cafe7d29f85f52fc2. PRISMA Extension for Scoping Reviews (PRISMA-ScR) [96] was followed as the reporting guideline.

### Eligibility criteria

Peer-reviewed, original research published in English, including the development and/or evaluation of LLMs in oncology-related aspects, for example (but not limited to), studies on cancer patient data, cancer literature data, and cancer education data were considered. To elaborate, development here refers to training or adapting an LLM (by changing its weights) for validating the same in cancer-related research question(s) using a dataset of more than one sample. Evaluation means validating an already trained LLM in cancer-related research question(s) using a dataset of more than one sample. These are the primary inclusion criteria. Exclusion criteria includes: article types such as commentaries, correspondences, reviews, surveys, abstracts, editorials, case reports, opinions, and pre-prints and publications in which the use of LLMs was not evident. Studies in which cancer/tumor were not in focus and different named pathologies were considered, were excluded as ‘Assessed as having reduced focus on cancer’. No restriction was placed on the nature of the LLMs’ application mentioned in the study if it involved the development and/or evaluation of LLMs for oncology.

### Information sources and search strategy

A librarian-assisted search was performed across the following databases: Association for Computing Machinery (ACM), Embase, Engineering Village, IEEE Xplore, Medline, Scopus, SPIE and Web of Science. The details of the search strategy can be found in our S1 Data workbook (tab: ‘Search Details’). The dates of coverage included from inception up until Jan 12, 2024. Pre-prints in this range were included only in the review if they were published/accepted in peer-reviewed venues by the cut-off date, set as Feb 29, 2024. Grey literature was not considered. Authors of selected articles were contacted if the use of LLM was not apparent in the study at the stage of full-text screening.

### Selection of the sources of evidence

The title and abstracts of the studies retrieved were uploaded to Covidence [97]. After deduplication, two independent reviewers (NM and TDD) conducted the title and abstract screening, followed by full text screening. Disagreements encountered in screening were resolved by a third reviewer (AS). A pilot screening process using 100 articles was performed before final screening to ensure that the screeners were being consistent with the set protocol. Research letters, letter to the editor, and short communication articles were considered on a case-by-case basis and included only if a defined methodology (related to development and/or evaluation) and detailed results were present.

### Data charting process

Data extraction was conducted using a pre-developed form in Covidence [97], created by consensus of all authors prior to the extraction process. This form was piloted for data extraction on 10 studies to ensure consistency and accuracy. For the majority of the questions, two reviewers (NM and TDD) independently extracted data from the included studies resulting from the full-text review, with each extraction being confirmed by the other reviewer (NM or TDD, as applicable) before being marked for completion. For optimization and evaluation-related questions, AS performed the extraction which was confirmed by other reviewers (NM and TDD). Any disagreements encountered were resolved by the discussion of all three reviewers to reach consensus. The items charted are presented in Table 4.

**Table 4:** Data items charted and their relevance to the primary and secondary questions studied.

Name of the item	Utilization
Title	General information related to the publication
Study ID and Citation	
Country of the Corresponding Author	
Year of Publication	
Venue of Publication	
Summary of the application	Pertinent to the first question and a secondary question. Coded to identify the primary focus of the application.
Cancer Type	
Nature of application	
User of the Application	
Phase of Cancer Care	Pertinent to the second question
Name and version of LLM evaluated or developed	Pertinent to the third question
Justification for choosing the LLM(s)	
Number of parameter(s) of the LLM involved	
Type of LLM	
Sources of Data used in development and/or evaluation	Pertinent to the fourth question
Preprocessing of data	
Demographics related to the dataset	
Demographics/information of users/human evaluators	
Development and Evaluation dataset size	
Techniques used to optimize LLMs (Does not include prompt-designing)	Pertinent to the fifth question
Evaluation Methodology (including metrics)	Pertinent to the sixth question
Limitation	Pertinent to the seventh question
Language supported by the application	Pertinent to a secondary question
Consideration of equity bias and socioeconomical differences in users	Pertinent to a secondary question

### Synthesis and presentation of results

A PRISMA flow diagram was constructed to demonstrate the search and screening processes. The data charted was used to answer seven primary questions:

### What types of applications are powered by LLMs?

The primary application(s) of the study was/were charted and categorized as developmental or fully evaluation-based on the usage of the LLM(s): (A) Developmental - proposal of a new LLM-based model architecture, a change in the model architecture made for adaptation [98], or pre-training and/or fine-tuning of existing architectures for specific tasks resulting changing weights of the LLMs (B) Evaluation – LLMs used off-the-shelf with or without prompt-engineering, i.e., the primary LLM architecture and weights were frozen from a previous pre-training as a part of a research question or used for feature representation. The cancer type(s) mentioned in relation to the application (particularly, when a model was evaluated) and the potential primary users of the application were collected from the studies or inferred from the context of the application.

### Which phases of cancer care are mostly addressed by LLMs?

The author-provided data on the utility of the application in a particular cancer phase was collected. The phases of cancer care are prevention, screening, diagnosis (i.e., detection in symptomatic individuals or individuals with concerning screening results), treatment, end-of-life, survivorship, and integrated care as per the Canadian Cancer Society guidelines [99]. If the exact matching phase(s) were not reported, we mapped the applications with the closest phase(s). Certain phases are difficult to distinguish unless clearly stated. This could happen particularly for prevention and screening and for screening and diagnosis. A study was considered as screening-only if screening was mentioned. In absence of any phase-related information, the data sources for evaluation were considered to derive the phase. In absence of authors’ reports or in case of failure to deduce the exact phases, the phase was considered ‘Non-specified’.

### Why and which LLMs (their characteristics) are being employed for development and evaluation tasks in oncology?

The LLM-driven software/interface or LLMs’ names and versions (when provided) were collected. The reasons stated for selecting or developing specific LLMs were collected and summarized.

### How are datasets being acquired and pre-processed for these tasks and what are the demographic characteristics?

The sources of the datasets used in development and evaluation were collected and re-coded into applicable categories. Furthermore, the demographic characteristics of the source datasets were collected. Demographic characteristics and qualifications of the human population involved in preparing the ground truth or evaluating the datasets were noted. The pre-processing steps applied to the data before using those as inputs to the LLMs or LLM-based software/interface were collected and summarized.

### What techniques are used to optimize LLMs?

In studies that were developmental in nature, technical details (including the data source, model, and comparative performance to existing models) to optimize the performance of the LLMs were collected and summarized.

### What are the methods of performance evaluation?

The measures applied to evaluate the performance in the primary study application were collected and grouped to simplify the nature of the performance evaluation. The grouping was informed by the charted data and was not pre-defined.

### What are the limitations noted by the authors and are there commonalities in the limitations?

The limitations mentioned in the studies were charted as a text summary. Subsequently, a thematic analysis was performed using QDA Miner Lite, a free computer-assisted qualitative analysis software [100]. The process involved coding each study and identifying sub-themes which were then developed into key parent themes (also called themes).

### Secondary research questions

We charted data pertaining to the following research questions: (a) Is genetic counseling a dominant application of LLMs? If yes, in which countries? (b) Other than the English, which languages were supported? (c) Are equity and socioeconomic differences in patients considered by LLMs? The first question was motivated by the usage of Chatbot in genetic cancer risk assessment and counseling [101]. The second question was motivated by prior finding that the English language is often the most supported language in natural language processing applications [102]. The third question is motivated by the fact that the datasets may not be reflective of the true patient population and need to be expanded to include equity, diversity and inclusion concepts. A recent study by Caglayan et al. [103] also supports this for oncology.

## Supporting information

S1 Data Indicating the search process, data charted, thematic analysis, funding sources of included studies, detailed analysis on evaluation. (XLSX)

S1 Checklist Document showing compliance with PRISMA-ScR. (DOCX)
