# Supplementary material for: Development and evaluation of large-language models (LLMs) for oncology: A scoping review
Source: PLOS Digit Health. 2025 Aug 7;4(8):e0000980. doi: 10.1371/journal.pdig.0000980 (PMC12331086; doi:10.1371/journal.pdig.0000980)
Supplement: S1 Checklist — (DOCX) [file pdig.0000980.s002.docx]

**Table 1 Compliance with PRISMA-ScR**

| **SECTION** | **ITEM** | **PRISMA-ScR CHECKLIST ITEM** | **DESCRIPTION OF COMPLIANCE** |
| --- | --- | --- | --- |
| **TITLE** | | | |
| Title | 1 | Identify the report as a scoping review. | The title of the report confirms its identification as a scoping review. |
| **ABSTRACT** | | | |
| Structured summary | 2 | Provide a structured summary that includes (as applicable): background, objectives, eligibility criteria, sources of evidence, charting methods, results, and conclusions that relate to the review questions and objectives. | A structured summary of the review is included under the abstract section. |
| **INTRODUCTION** | | | |
| Rationale | 3 | Describe the rationale for the review in the context of what is already known. Explain why the review questions/objectives lend themselves to a scoping review approach. | A rationale is provided as part of the introduction section of the report. (Refer to 'Background and Rationale' under 'Introduction') |
| Objectives | 4 | Provide an explicit statement of the questions and objectives being addressed with reference to their key elements (e.g., population or participants, concepts, and context) or other relevant key elements used to conceptualize the review questions and/or objectives. | A statement of objectives and questions being addressed is included under the Introduction section. (Refer to last pargraph under 'Introduction') |
| **METHODS** | | | |
| Protocol and registration | 5 | Indicate whether a review protocol exists; state if and where it can be accessed (e.g., a Web address); and if available, provide registration information, including the registration number. | A review protocol along with a link to the access is included in the 'Materials and Methods' section. (Refer to 'Study Framework, Protocol and Reporting' under 'Materials and Methods') |
| Eligibility criteria | 6 | Specify characteristics of the sources of evidence used as eligibility criteria (e.g., years considered, language, and publication status), and provide a rationale. | The eligibility criteria for included studies is reported under the 'Materials and Methods' section. (Refer to 'Eligibility Criteria' under 'Materials and Methods') |
| Information sources* | 7 | Describe all information sources in the search (e.g., databases with dates of coverage and contact with authors to identify additional sources), as well as the date the most recent search was executed. | Information sources with the date of the most recent search are included in the Methods section (Refer to 'Information Sources and Search Strategy' under 'Materials and Methods') |
| Search | 8 | Present the full electronic search strategy for at least 1 database, including any limits used, such that it could be repeated. | The search strategy for all databases are provided in supplementary data under Table S1: Search Criteria used in our Study (Refer to tab 'Search Details'. i.e., Sheet 1, Supplementary data workbook) |
| Selection of sources of evidence† | 9 | State the process for selecting sources of evidence (i.e., screening and eligibility) included in the scoping review. | The selection process for study inclusion is reported in the Methods section. (Refer to 'Selection of the Sources of Evidence' under 'Materials and Methods') |
| Data charting process‡ | 10 | Describe the methods of charting data from the included sources of evidence (e.g., calibrated forms or forms that have been tested by the team before their use, and whether data charting was done independently or in duplicate) and any processes for obtaining and confirming data from investigators. | The data charting process is outlined in the Materials and Methods section. (Refer to 'Data Charting Process' under 'Materials and Methods') |
| Data items | 11 | List and define all variables for which data were sought and any assumptions and simplifications made. | Data Items are listed and defined in Table 1. (Refer to Table 1 under 'Materials and Methods') |
| Critical appraisal of individual sources of evidence§ | 12 | If done, provide a rationale for conducting a critical appraisal of included sources of evidence; describe the methods used and how this information was used in any data synthesis (if appropriate). | A critical appraisal of included sources was not performed and is noted as a limitation of the report. |
| Synthesis of results | 13 | Describe the methods of handling and summarizing the data that were charted. | Methods for handling and summarizing data for each objective/question are reported under the Methods section. (Refer to 'Synthesis and Presentation of Results' under 'Materials and Methods') |
| **RESULTS** | | | |
| Selection of sources of evidence | 14 | Give numbers of sources of evidence screened, assessed for eligibility, and included in the review, with reasons for exclusions at each stage, ideally using a flow diagram. | Numbers of sources of evidence are included under the Results section. (Refer to 'Selection of Sources of Evidence' under 'Results'). The flow diagram is included as Fig.1. |
| Characteristics of sources of evidence | 15 | For each source of evidence, present characteristics for which data were charted and provide the citations. | Data Items are listed and defined in supplementary data under Table S2: Data Charted. (Refer to tab: 'Data Charted', i.e., Sheet 2 and ‘Evaluation-Detailed Analysis’ i.e., Sheet 5, Supplementary data workbook) and in Table 2 in main manuscript. All the sources are cited within the 'Results' section of the manuscript. |
| Critical appraisal within sources of evidence | 16 | If done, present data on critical appraisal of included sources of evidence (see item 12). | A critical appraisal of included sources was not performed and is noted as a limitation of the report. |
| Results of individual sources of evidence | 17 | For each included source of evidence, present the relevant data that were charted that relate to the review questions and objectives. | Data Items are listed and defined in supplementary data under Table S2: Data Charted. (Refer to tab: 'Data Charted', i.e., Sheet 2, and ‘Evaluation-Detailed Analysis’ i.e., Sheet 5, Supplementary data workbook) and in Table 2 in main manuscript. |
| Synthesis of results | 18 | Summarize and/or present the charting results as they relate to the review questions and objectives. | The charting results are reported for each objective/question under the Results section. |
| **DISCUSSION** | | | |
| Summary of evidence | 19 | Summarize the main results (including an overview of concepts, themes, and types of evidence available), link to the review questions and objectives, and consider the relevance to key groups. | The main results of the report are presented under the Discussion section. |
| Limitations | 20 | Discuss the limitations of the scoping review process. | Limitations of the review are provided in the Discussion section. (Refer to the last paragraph under 'Discussion') |
| Conclusions | 21 | Provide a general interpretation of the results with respect to the review questions and objectives, as well as potential implications and/or next steps. | An interpretation of results along with implications on research and practice are included under the Conclusions section. |
| **FUNDING** | | | |
| Funding | 22 | Describe sources of funding for the included sources of evidence, as well as sources of funding for the scoping review. Describe the role of the funders of the scoping review. | Funding sources for all included studies are provided in Table S4: Funding Sources of Included Sources of Evidence. (Refer to Sheet 4, Supplementary data workbook). The funding sources of our review is provided (none) in the 'Financial Disclosure Statement' of the manuscript. |
| JBI = Joanna Briggs Institute; PRISMA-ScR = Preferred Reporting Items for Systematic reviews and Meta-Analyses extension for Scoping Reviews. | | | |
| * Where *sources of evidence* (see second footnote) are compiled from, such as bibliographic databases, social media platforms, and Web sites. | | | |
| † A more inclusive/heterogeneous term used to account for the different types of evidence or data sources (e.g., quantitative and/or qualitative research, expert opinion, and policy documents) that may be eligible in a scoping review as opposed to only studies. This is not to be confused with *information sources* (see first footnote). | | | |
| ‡ The frameworks by Arksey and O’Malley (6) and Levac and colleagues (7) and the JBI guidance (4, 5) refer to the process of data extraction in a scoping review as data charting*.* | | | |
| § The process of systematically examining research evidence to assess its validity, results, and relevance before using it to inform a decision. This term is used for items 12 and 19 instead of "risk of bias" (which is more applicable to systematic reviews of interventions) to include and acknowledge the various sources of evidence that may be used in a scoping review (e.g., quantitative and/or qualitative research, expert opinion, and policy document). | | | |
|  |  |  |  |
|  |  |  |  |
| [From: Tricco AC, Lillie E, Zarin W, O'Brien KK, Colquhoun H, Levac D, et al. PRISMA Extension for Scoping Reviews (PRISMAScR): Checklist and Explanation. Ann Intern Med. 2018;169:467–473. doi: 10.7326/M18-0850.](http://annals.org/aim/fullarticle/2700389/prisma-extension-scoping-reviews-prisma-scr-checklist-explanation) | | | |
